# Supplementary material for: Acupuncture therapies for relieving pain in pelvic inflammatory disease: A systematic review and meta-analysis
Source: PLoS One. 2024 Jan 31;19(1):e0292166. doi: 10.1371/journal.pone.0292166 (PMC10830011; doi:10.1371/journal.pone.0292166)
Supplement: S1 Appendix — (DOCX) [file pone.0292166.s002.docx]

**Appendix**

**Pubmed**

| **1#** | **((((((((((((((((((****Pelvic Inflammatory Disease[MeSH Terms]) OR (Disease, Pelvic Inflammatory[Title/Abstract])) OR (Diseases, Pelvic Inflammatory[Title/Abstract])) OR (Inflammatory Diseases, Pelvic[Title/Abstract])) OR (Pelvic Inflammatory Diseases[Title/Abstract])) OR (Inflammatory Pelvic Disease[Title/Abstract])) OR (Disease, Inflammatory Pelvic[Title/Abstract])) OR (Diseases, Inflammatory Pelvic[Title/Abstract])) OR (Inflammatory Pelvic Diseases[Title/Abstract])) OR (Pelvic Diseases, Inflammatory[Title/Abstract])) OR (Pelvic Disease, Inflammatory[Title/Abstract])) OR (Inflammatory Disease, Pelvic[Title/Abstract])) OR (Adnexitis[Title/Abstract])) OR (p.i.d.[Title/Abstract])) OR (pelvic infection[Title/Abstract])) OR (pelvic inflammation[Title/Abstract])) OR (pelvis infection[Title/Abstract])) OR (pelvis inflammation[Title/Abstract])) OR (pelvis inflammatory disease[Title/Abstract])** |
| --- | --- |
| **2#** | **(Endometritis[MeSH Terms]) OR (Endomyometritis[Title/Abstract])** |
| **3#** | **(((((((((((Salpingitis[MeSH Terms]) OR (Salpingitides[Title/Abstract])) OR (acute salpingitis[Title/Abstract])) OR (fallopian tubal inflammation[Title/Abstract])) OR (fallopian tubal inflammatory disease[Title/Abstract])) OR (fallopian tube inflammation[Title/Abstract])) OR (oviduct inflammation[Title/Abstract])) OR (oviductal inflammation[Title/Abstract])) OR (salpingeal inflammation[Title/Abstract])) OR (salpingitis, acute[Title/Abstract])) OR (uterine tubal inflammation[Title/Abstract])) OR (uterine tube inflammation[Title/Abstract])** |
| **4#** | **((((((Oophoritis[MeSH Terms]) OR (Oophoritides[Title/Abstract])) OR (ovary inflammation[Title/Abstract])) OR (inflammatio ovarica[Title/Abstract])) OR (inflammation, ovary[Title/Abstract])) OR (ovarial inflammation[Title/Abstract])) OR (ovaritis[Title/Abstract])** |
| **5#** | **((((((((((((Parametritis[MeSH Terms]) OR (Parametritides[Title/Abstract])) OR (Cellulitis, Pelvic[Title/Abstract])) OR (Cellulitides, Pelvic[Title/Abstract])) OR (Pelvic Cellulitides[Title/Abstract])) OR (Pelvic Cellulitis[Title/Abstract])) OR (adnexitis[Title/Abstract])) OR (adnex inflammation, uterine[Title/Abstract])) OR (adnexa infection[Title/Abstract])) OR (adnexa inflammation[Title/Abstract])) OR (adnexitis, uterine[Title/Abstract])) OR (para metriosis[Title/Abstract])) OR (uterine adnexitis[Title/Abstract])** |
| **6#** | **1# OR 2# OR 3# OR 4# OR 5#** |
| **7#** | **((((acupuncture[MeSH Terms]) OR (****acupuncture therapy[MeSH Terms])) OR (****acupuncture,ear[MeSH Terms])) OR (****acupuncture points[MeSH Terms])) OR (****Meridians[MeSH Terms])** |
| **8#** | **(((((((((((((((Acupuncture Treatment[Title/Abstract]) OR (Acupuncture Treatments[Title/Abstract])) OR (Treatment, Acupuncture[Title/Abstract])) OR (Therapy, Acupuncture[Title/Abstract])) OR (Treatment, Acupotomy[Title/Abstract])) OR (Acupotomies[Title/Abstract])) OR (herb acupuncture[Title/Abstract])) OR (herbal acupuncture[Title/Abstract])) OR (herbalized acupuncture[Title/Abstract])) OR (Pharmacopuncture[Title/Abstract])) OR (pharmaco-acupuncture[Title/Abstract])) OR (pharmaco-puncture[Title/Abstract])) OR (Pharmacoacupuncture[Title/Abstract])) OR (Pharmacoacupuncture Therapy[Title/Abstract])) OR (Therapy, Pharmacoacupuncture[Title/Abstract])) OR (Pharmacoacupuncture Treatment[Title/Abstract])** |
| **9#** | **(((((((((((Acupunctures, Ear[Title/Abstract]) OR (acupuncture, earlobe[Title/Abstract])) OR (earlobe acupuncture[Title/Abstract])) OR (auriculotherapy[Title/Abstract])) OR (Ear Acupunctures[Title/Abstract])) OR (Ear Acupuncture[Title/Abstract])) OR (Auricular Acupunctures[Title/Abstract])) OR (Auricular Acupuncture[Title/Abstract])) OR (Acupuncture, Auricular[Title/Abstract])) OR (Acupunctures, Auricular[Title/Abstract])) OR (auriculo-acupuncture[Title/Abstract])) OR (auriculoacupuncture[Title/Abstract])** |
| **10#** | **(((((((acupuncture, electric[Title/Abstract]) OR (electrical acupoint stimulation[Title/Abstract])) OR (Electroacupuncture[Title/Abstract])) OR (electric acupuncture[Title/Abstract])) OR (electrical acupuncture[Title/Abstract])) OR (electronic acupuncture[Title/Abstract])) OR (electrode acupuncture[Title/Abstract])) OR (electro-acupuncture[Title/Abstract])** |
| **11#** | **(((((Acupuncture Point[Title/Abstract]) OR (acu-point[Title/Abstract])) OR (Acupoints[Title/Abstract])) OR (Acupoint[Title/Abstract])) OR (Point, Acupuncture[Title/Abstract])) OR (Points, Acupuncture[Title/Abstract])** |
| **12#** | **((((((((((((((warming needle moxibustion[Title/Abstract]) OR (needle warming moxibustion[Title/Abstract])) OR (burnt needle therapy[Title/Abstract])) OR (warm needle acupuncture[Title/Abstract])) OR (fire acupuncture[Title/Abstract])) OR (fire needle acupuncture[Title/Abstract])) OR (fire needle[Title/Abstract])) OR (fire needling[Title/Abstract])) OR (fire needle therapy[Title/Abstract])) OR (fire needle moxibustion[Title/Abstract])) OR (heat acupuncture[Title/Abstract])) OR (heat-type acupuncture[Title/Abstract])) OR (thermal acupuncture[Title/Abstract])) OR (thermo-acupuncture[Title/Abstract])) OR (thermoacupuncture[Title/Abstract])** |
| **13#** | **((((catgut embedding[Title/Abstract]) OR (catgut embedding therapy[Title/Abstract])) OR (ACET[Title/Abstract])) OR (acupoint catgut embedding therapy[Title/Abstract])) OR (catgut implantation[Title/Abstract])** |
| **14#** | **((((((Ching Lo[Title/Abstract]) OR (Jing Luo[Title/Abstract])) OR (Luo, Jing[Title/Abstract])) OR (Jingluo[Title/Abstract])) OR (meridian system[Title/Abstract])) OR (meridian network[Title/Abstract])) OR (meridian-collateral system[Title/Abstract])** |
| **15#** | **((((((hydro-acupuncture[Title/Abstract]) OR (scalp acupuncture[Title/Abstract])) OR (Abdominal acupuncture[Title/Abstract])) OR (Fu's subcutaneous needling therapy[Title/Abstract])) OR (Fu's acupuncture[Title/Abstract])) OR (floating needle[Title/Abstract])) OR (float needle[Title/Abstract])** |
| **16#** | **7# OR 15#** |
| **17#** | **((((((Controlled Clinical Trial [Publication Type]) OR (Randomized Controlled Trial [Publication Type])) OR (randomized[Title/Abstract])) OR (placebo[Title/Abstract])) OR (randomly[Title/Abstract])) OR (trial[Title/Abstract])) OR (groups[Title/Abstract])** |
| **18#** | **6# AND 16# AND 17#** |

**Embase**

| #1 | 'pelvic inflammatory disease'/exp |
| --- | --- |
| #2 | 'disease, pelvic inflammatory':ab,ti OR 'diseases, pelvic inflammatory':ab,ti OR 'inflammatory diseases, pelvic':ab,ti OR 'pelvic inflammatory diseases':ab,ti OR 'inflammatory pelvic disease':ab,ti OR 'disease, inflammatory pelvic':ab,ti OR 'diseases, inflammatory pelvic':ab,ti OR 'inflammatory pelvic diseases':ab,ti OR 'pelvic diseases, inflammatory':ab,ti OR 'pelvic disease, inflammatory':ab,ti OR 'inflammatory disease, pelvic':ab,ti OR adnexitis:ab,ti OR p.i.d.:ab,ti OR 'pelvic infection':ab,ti OR 'pelvic inflammation':ab,ti OR 'pelvis infection':ab,ti OR 'pelvis inflammation':ab,ti OR 'pelvis inflammatory disease':ab,ti |
| #3 | #1 OR #2 |
| #4 | 'endometritis'/exp |
| #5 | endomyometritis:ab,ti |
| #6 | #4 OR #5 |
| #7 | 'salpingitis'/exp |
| #8 | salpingitides:ab,ti OR 'acute salpingitis':ab,ti OR 'fallopian tubal inflammation':ab,ti OR 'fallopian tubal inflammatory disease':ab,ti OR 'fallopian tube inflammation':ab,ti OR 'oviduct inflammation':ab,ti OR 'oviductal inflammation':ab,ti OR 'salpingeal inflammation':ab,ti OR 'salpingitis, acute':ab,ti OR 'uterine tubal inflammation':ab,ti OR 'uterine tube inflammation':ab,ti |
| #9 | #7 OR #8 |
| #10 | 'ovary inflammation'/exp |
| #11 | oophoritis:ab,ti OR oophoritides:ab,ti OR 'inflammatio ovarica':ab,ti OR 'inflammation, ovary':ab,ti OR 'ovarial inflammation':ab,ti OR ovaritis:ab,ti |
| #12 | #10 OR #11 |
| #13 | 'adnexitis'/exp |
| #14 | parametritis:ab,ti OR parametritides:ab,ti OR 'cellulitis, pelvic':ab,ti OR 'cellulitides, pelvic':ab,ti OR 'pelvic cellulitides':ab,ti OR 'pelvic cellulitis':ab,ti OR 'adnex inflammation, uterine':ab,ti OR 'adnexa infection':ab,ti OR 'adnexa inflammation':ab,ti OR 'adnexitis, uterine':ab,ti OR 'para metriosis':ab,ti OR 'uterine adnexitis':ab,ti |
| #15 | #13 OR #14 |
| #16 | #3 OR #6 OR #9 OR #12 OR #15 |
| #17 | 'acupuncture'/exp |
| #18 | 'auricular acupuncture'/exp |
| #19 | 'catgut embedding'/exp |
| #20 | 'electroacupuncture'/exp |
| #21 | 'pharmacopuncture'/exp |
| #22 | 'warm acupuncture'/exp |
| #23 | 'acupuncture point'/exp |
| #24 | 'warm acupuncture'/exp |
| #25 | #17 OR #18 OR #19 OR #20 OR #21 OR #22 OR #23 OR #24 |
| #26 | 'acupuncture therapy':ab,ti OR shonishin:ab,ti OR 'herb acupuncture':ab,ti OR 'herbal acupuncture':ab,ti OR 'herbalized acupuncture':ab,ti OR 'pharmaco acupuncture':ab,ti OR 'pharmaco puncture':ab,ti OR pharmacoacupuncture:ab,ti OR 'acupuncture treatment':ab,ti OR 'acupuncture treatments':ab,ti OR 'treatment, acupuncture':ab,ti OR 'therapy, acupuncture':ab,ti OR 'pharmacoacupuncture treatment':ab,ti OR 'treatment, pharmacoacupuncture':ab,ti OR 'pharmacoacupuncture therapy':ab,ti OR 'therapy, pharmacoacupuncture':ab,ti OR acupotomy:ab,ti OR acupotomies:ab,ti |
| #27 | 'acupuncture, ear':ab,ti OR 'acupuncture, earlobe':ab,ti OR 'auriculo acupuncture':ab,ti OR auriculoacupuncture:ab,ti OR auriculotherapy:ab,ti OR 'ear acupuncture':ab,ti OR 'earlobe acupuncture':ab,ti OR 'acupunctures, ear':ab,ti OR 'ear acupunctures':ab,ti OR 'auricular acupuncture':ab,ti OR 'acupunctures, auricular':ab,ti OR 'auricular acupunctures':ab,ti |
| #28 | acet:ab,ti AND 'acupoint catgut embedding therapy':ab,ti OR 'acupoint catgut embedding therapy':ab,ti OR 'catgut implantation':ab,ti OR 'catgut embedding therapy':ab,ti |
| #29 | 'acupuncture, electric':ab,ti OR 'electric acupuncture':ab,ti OR 'electrical acupoint stimulation':ab,ti OR 'electrical acupuncture':ab,ti OR 'electro acupuncture':ab,ti OR 'electrode acupuncture':ab,ti OR 'electronic acupuncture':ab,ti |
| #30 | 'burnt needle therapy':ab,ti OR 'fire acupuncture':ab,ti OR 'fire needle acupuncture':ab,ti OR 'fire needle therapy':ab,ti OR 'fire needling':ab,ti OR 'heat acupuncture':ab,ti OR 'heat-type acupuncture':ab,ti OR 'thermal acupuncture':ab,ti OR 'thermo acupuncture':ab,ti OR thermoacupuncture:ab,ti OR 'warm needle acupuncture':ab,ti OR 'warming needle moxibustion':ab,ti OR 'needle warming moxibustion':ab,ti OR 'fire needle':ab,ti OR 'fire needle moxibustion':ab,ti |
| #31 | 'acu point':ab,ti OR acupoint:ab,ti OR acupoints:ab,ti OR 'acupuncture points':ab,ti OR 'point, acupuncture':ab,ti OR 'points, acupuncture':ab,ti |
| #32 | 'jing luo':ab,ti OR jingluo:ab,ti OR 'meridian network':ab,ti OR 'meridian-collateral system':ab,ti OR 'ching lo':ab,ti OR 'luo, jing':ab,ti OR meridians:ab,ti |
| #33 | 'hydro acupuncture':ab,ti OR 'scalp acupuncture':ab,ti OR 'fus subcutaneous needling therapy':ab,ti OR 'fus acupuncture':ab,ti OR 'floating needle':ab,ti OR 'float needle':ab,ti OR 'abdominal acupuncture':ab,ti |
| #34 | #26 OR #27 OR #28 OR #29 OR #30 OR #31 OR #32 OR #33 |
| #35 | #25 OR #34 |
| #36 | 'randomized controlled trial'/exp |
| #37 | 'controlled clinical trial'/exp |
| #38 | randomized:ab,ti OR placebo:ab,ti OR randomly:ab,ti OR trial:ab,ti OR groups:ab,ti |
| #39 | #36 OR #37 OR #38 |
| #40 | #16 AND #35 AND #39 |

**Cochrane**

| #1 | MeSH descriptor: [Pelvic Inflammatory Disease] explode all trees |
| --- | --- |
| #2 | MeSH descriptor: [Endometritis] explode all trees |
| #3 | MeSH descriptor: [Salpingitis] explode all trees |
| #4 | MeSH descriptor: [Oophoritis] explode all trees |
| #5 | MeSH descriptor: [Parametritis] explode all trees |
| #6 | (Pelvic Inflammatory Disease OR Disease, Pelvic Inflammatory OR Diseases, Pelvic Inflammatory OR Inflammatory Diseases, Pelvic OR Pelvic Inflammatory Diseases OR Inflammatory Pelvic Disease OR Disease, Inflammatory Pelvic OR Diseases, Inflammatory Pelvic OR Inflammatory Pelvic Diseases OR Pelvic Diseases, Inflammatory OR Pelvic Disease, Inflammatory OR Inflammatory Disease, Pelvic OR Adnexitis OR p.i.d. OR pelvic infection OR pelvic inflammation OR pelvis infection OR pelvis inflammation OR pelvis inflammatory disease):ti,ab,kw (Word variations have been searched) |
| #7 | (Endometritis OR Endomyometritis):ti,ab,kw (Word variations have been searched) |
| #8 | (Salpingitis OR Salpingitides OR acute salpingitis OR fallopian tubal inflammation OR fallopian tubal inflammatory disease OR fallopian tube inflammation OR oviduct inflammation OR oviductal inflammation OR salpingeal inflammation OR salpingitis, acute OR uterine tubal inflammation OR uterine tube inflammation):ti,ab,kw (Word variations have been searched) |
| #9 | (Oophoritis OR Oophoritides OR ovary inflammation OR inflammatio ovarica OR inflammation, ovary OR ovarial inflammation OR ovaritis):ti,ab,kw (Word variations have been searched) |
| #10 | (Parametritis OR Parametritides OR Cellulitis, Pelvic OR Cellulitides, Pelvic OR Pelvic Cellulitides OR Pelvic Cellulitis OR adnexitis OR adnex inflammation, uterine OR adnexa infection OR adnexa inflammation OR adnexitis, uterine OR para metriosis OR uterine adnexitis):ti,ab,kw (Word variations have been searched) |
| #11 | #1 OR #2 OR #3 OR #4 OR #5 OR #6 OR #7 OR #8 OR #9 OR #10 |
| #12 | MeSH descriptor: [Acupuncture] explode all trees |
| #13 | MeSH descriptor: [Acupuncture Therapy] explode all trees |
| #14 | MeSH descriptor: [Acupuncture, Ear] explode all trees |
| #15 | MeSH descriptor: [Meridians] explode all trees |
| #16 | (Acupuncture Treatment OR Acupuncture Treatments OR Treatment, Acupuncture OR Therapy, Acupuncture OR Treatment, Acupotomy OR Acupotomies OR herb acupuncture OR herbal acupuncture OR herbalized acupuncture OR Pharmacopuncture OR pharmaco-acupuncture OR pharmaco-puncture OR Pharmacoacupuncture OR Pharmacoacupuncture Therapy OR Therapy, Pharmacoacupuncture OR Pharmacoacupuncture Treatment):ti,ab,kw (Word variations have been searched) |
| #17 | (Acupunctures, Ear OR acupuncture, earlobe OR earlobe acupuncture OR auriculotherapy OR Ear Acupunctures OR Ear Acupuncture OR Auricular Acupunctures OR Auricular Acupuncture OR Acupuncture, Auricular OR Acupunctures, Auricular OR auriculo-acupuncture OR auriculoacupuncture):ti,ab,kw (Word variations have been searched) |
| #18 | (acupuncture, electric OR electrical acupoint stimulation OR Electroacupuncture OR electric acupuncture OR electrical acupuncture OR electronic acupuncture OR electrode acupuncture OR electro-acupuncture):ti,ab,kw (Word variations have been searched) |
| #19 | (Acupuncture Point OR acu-point OR Acupoints OR Acupoint OR Point, Acupuncture OR Points, Acupuncture):ti,ab,kw (Word variations have been searched) |
| #20 | (warming needle moxibustion OR needle warming moxibustion OR burnt needle therapy OR warm needle acupuncture OR fire acupuncture OR fire needle acupuncture OR fire needle OR fire needling OR fire needle therapy OR fire needle moxibustion OR heat acupuncture OR heat-type acupuncture OR thermal acupuncture OR thermo-acupuncture OR thermoacupuncture):ti,ab,kw (Word variations have been searched) |
| #21 | (catgut embedding OR catgut embedding therapy OR ACET OR acupoint catgut embedding therapy OR catgut implantation):ti,ab,kw (Word variations have been searched) |
| #22 | (Ching Lo OR Jing Luo OR Luo, Jing OR Jingluo OR meridian system OR meridian network OR meridian-collateral system):ti,ab,kw (Word variations have been searched) |
| #23 | (hydro-acupuncture OR scalp acupuncture OR Abdominal acupuncture OR Fu's subcutaneous needling therapy OR Fu's acupuncture OR floating needle OR float needle):ti,ab,kw (Word variations have been searched) |
| #24 | #12 OR #13 OR #14 OR #15 OR #16 OR #17 OR #18 OR #19 OR #20 OR #21 OR #22 OR #23 |
| #25 | MeSH descriptor: [Randomized Controlled Trial] explode all trees |
| #26 | MeSH descriptor: [Controlled Clinical Trial] explode all trees |
| #27 | (Controlled Clinical Trial OR Randomized Controlled Trial OR randomized OR placebo OR randomly OR trial OR groups):ti,ab,kw (Word variations have been searched) |
| #28 | #25 OR #26 OR #27 |
| #29 | #11 AND #24 AND #28 |

**Web of Science**

| #1 | TS=(Pelvic Inflammatory Disease OR Disease, Pelvic Inflammatory OR Diseases, Pelvic Inflammatory OR Inflammatory Diseases, Pelvic OR Pelvic Inflammatory Diseases OR Inflammatory Pelvic Disease OR Disease, Inflammatory Pelvic OR Diseases, Inflammatory Pelvic OR Inflammatory Pelvic Diseases OR Pelvic Diseases, Inflammatory OR Pelvic Disease, Inflammatory OR Inflammatory Disease, Pelvic OR Adnexitis OR p.i.d. OR pelvic infection OR pelvic inflammation OR pelvis infection OR pelvis inflammation OR pelvis inflammatory disease) |
| --- | --- |
| #2 | **TS=(Endometritis OR Endomyometritis)** |
| #3 | **TS=(Salpingitis OR Salpingitides OR acute salpingitis OR fallopian tubal inflammation OR fallopian tubal inflammatory disease OR fallopian tube inflammation OR oviduct inflammation OR oviductal inflammation OR salpingeal inflammation OR salpingitis, acute OR uterine tubal inflammation OR uterine tube inflammation)** |
| #4 | **TS=(Oophoritis OR Oophoritides OR ovary inflammation OR inflammatio ovarica OR inflammation, ovary OR ovarial inflammation OR ovaritis)** |
| #5 | **TS=(Parametritis OR Parametritides OR Cellulitis, Pelvic OR Cellulitides, Pelvic OR Pelvic Cellulitides OR Pelvic Cellulitis OR adnexitis OR adnex inflammation, uterine OR adnexa infection OR adnexa inflammation OR adnexitis, uterine OR para metriosis OR uterine adnexitis)** |
| #6 | **#5 OR #4 OR #3 OR #2 OR #1** |
| #7 | **TS=(acupuncture OR acupuncture therapy OR acupuncture,ear OR acupuncture points OR Meridians)** |
| #8 | **TS=(Acupuncture Treatment OR Acupuncture Treatments OR Treatment, Acupuncture OR Therapy, Acupuncture OR Treatment, Acupotomy OR Acupotomies OR herb acupuncture OR herbal acupuncture OR herbalized acupuncture OR Pharmacopuncture OR pharmaco-acupuncture OR pharmaco-puncture OR Pharmacoacupuncture OR Pharmacoacupuncture Therapy OR Therapy, Pharmacoacupuncture OR Pharmacoacupuncture Treatment)** |
| #9 | **TS=(Acupunctures, Ear OR acupuncture, earlobe OR earlobe acupuncture OR auriculotherapy OR Ear Acupunctures OR Ear Acupuncture OR Auricular Acupunctures OR Auricular Acupuncture OR Acupuncture, Auricular OR Acupunctures, Auricular OR auriculo-acupuncture OR auriculoacupuncture)** |
| #10 | **TS=(****acupuncture, electric OR electrical acupoint stimulation OR Electroacupuncture OR electric acupuncture OR electrical acupuncture OR electronic acupuncture OR electrode acupuncture OR electro-acupuncture)** |
| #11 | **TS=(****Acupuncture Point OR acu-point OR Acupoints OR Acupoint OR Point, Acupuncture OR Points, Acupuncture)** |
| #12 | **TS=(****warming needle moxibustion OR needle warming moxibustion OR burnt needle therapy OR warm needle acupuncture OR fire acupuncture OR fire needle acupuncture OR fire needle OR fire needling OR fire needle therapy OR fire needle moxibustion OR heat acupuncture OR heat-type acupuncture OR thermal acupuncture OR thermo-acupuncture OR thermoacupuncture)** |
| #13 | **TS=(****catgut embedding OR catgut embedding therapy OR ACET OR acupoint catgut embedding therapy OR catgut implantation)** |
| #14 | **TS=(****Ching Lo OR Jing Luo OR Luo, Jing OR Jingluo OR meridian system OR meridian network OR meridian-collateral system)** |
| #15 | **TS=(****hydro-acupuncture OR scalp acupuncture OR Abdominal acupuncture OR Fu's subcutaneous needling therapy OR Fu's acupuncture OR floating needle OR float needle)** |
| #16 | **#7 OR #8 OR #9 OR #10 OR #11 OR #12 OR #13 OR #14 OR #15** |
| #17 | **TS=(****Controlled Clinical Trial OR Randomized Controlled Trial OR randomized OR placebo OR randomly OR trial OR groups)** |
| #18 | **#6 AND #16 AND #17** |

**CNKI**

(((主题%= 盆腔炎性疾病 + 子宫附件炎 + 子宫内膜炎 + 子宫内膜炎症 + 卵巢炎 + 子宫旁组织炎 + 盆腔峰窝织炎 + 输卵管炎 + 盆腔炎 or 题名%= 盆腔炎性疾病 + 子宫附件炎 + 子宫内膜炎 + 子宫内膜炎症 + 卵巢炎 + 子宫旁组织炎 + 盆腔蜂窝织炎 + 输卵管炎 + 盆腔炎) AND ((主题%= 针灸 + 针灸疗法 + 针刺疗法 + 针刺 + 针法 + 火针疗法 + 毫火针疗法 + 火针 + 毫火针 + 穴位注射 + 穴位注射疗法 + 微针疗法 + 耳针疗法 + 耳针 + 头针疗法 + 头针 + 浮针疗法 + 浮针 + 腹针疗法 or 题名%= 针灸 + 针灸疗法 + 针刺疗法 + 针刺 + 针法 + 火针疗法 + 毫火针疗法 + 火针 + 毫火针 + 穴位注射 + 穴位注射疗法 + 微针疗法 + 耳针疗法 + 耳针 + 头针疗法 + 头针 + 浮针疗法 + 浮针 + 腹针疗法) OR (主题%= 腹针 + 头皮针 + 体针疗法 + 体针 + 毫针 + 灸法 + 艾灸 + 温针 + 温针灸 + 温针疗法 + 隔物灸 + 穴位疗法 + 穴位埋线 + 埋线 + 耳穴 + 针药并用 or 题名%=腹针 + 头皮针 + 体针疗法 + 体针 + 毫针 + 灸法 + 艾灸 + 温针 + 温针灸 + 温针疗法 + 隔物灸 + 穴位疗法 + 穴位埋线 + 埋线 + 耳六 + 针药并用))) AND (主题%= 随机对照 + 随机 + 对照 + xls(RCT) + 临床研究 + 疗效观察 + 临床观察 + 疗效 + 观察 + 临床 or 题名%= 随机对照 + 随机 + 对照 + xls(RCT + 临床研究 + 疗效观察 + 临床观察 + 疗效 + 观察 + 临床))

**CBM**

| #1 | ((("盆腔炎性疾病"[不加权:扩展]) OR "子宫内膜炎"[不加权:扩展]) OR "卵巢炎"[不加权:扩展]) OR "输卵管炎"[不加权:扩展] |
| --- | --- |
| #2 | "子宫附件炎"[常用字段:智能] OR "子宫内膜炎症"[常用字段:智能] OR "盆腔蜂窝织炎"[常用字段:智能] OR "输卵管炎"[常用字段:智能] OR "盆腔炎"[常用字段:智能] |
| #3 | #1 OR #2 |
| #4 | (((((((((((((("针灸疗法"[不加权:扩展]) OR "针刺疗法"[不加权:扩展]) OR "灸法"[不加权:扩展]) OR "针药并用疗法"[不加权:扩展]) OR "穴位疗法"[不加权:扩展]) OR "火针疗法"[不加权:扩展]) OR "水针疗法"[不加权:扩展]) OR "微针疗法"[不加权:扩展]) OR "体针疗法"[不加权:扩展]) OR "电针疗法"[不加权:扩展]) OR "耳针疗法"[不加权:扩展]) OR "头针疗法"[不加权:扩展]) OR "浮针疗法"[不加权:扩展]) OR "腹针疗法"[不加权:扩展]) OR "温针疗法"[不加权:扩展] |
| #5 | "针灸"[常用字段:智能] OR "针法"[常用字段:智能] OR "针刺"[常用字段:智能] OR "毫火针疗法"[常用字段:智能] OR "火针"[常用字段:智能] OR "毫火针"[常用字段:智能] OR "穴位注射"[常用字段:智能] OR "穴位注射疗法"[常用字段:智能] OR "耳针"[常用字段:智能] OR "头针"[常用字段:智能] OR "头皮针"[常用字段:智能] OR "浮针"[常用字段:智能] OR "腹针"[常用字段:智能] OR "体针"[常用字段:智能] OR "毫针"[常用字段:智能] OR "艾灸"[常用字段:智能] OR "温针"[常用字段:智能] OR "温针灸"[常用字段:智能] OR "隔物灸"[常用字段:智能] OR "穴位埋线"[常用字段:智能] OR "埋线"[常用字段:智能] OR "耳穴"[常用字段:智能] |
| #6 | #4 OR #5 |
| #7 | "随机对照试验"[不加权:扩展] |
| #8 | "随机对照"[常用字段:智能] OR "随机"[常用字段:智能] OR "对照"[常用字段:智能] OR "临床观察"[常用字段:智能] OR "临床研究"[常用字段:智能] OR "疗效观察"[常用字段:智能] OR "疗效"[常用字段:智能] OR "观察"[常用字段:智能] OR "临床"[常用字段:智能] OR "RCT"[常用字段:智能] |
| #9 | #7 OR #8 |
| #10 | #3 AND #6 AND #9 |

**VIP**

((((((((((题名或关键词=盆腔炎性疾病 OR 题名或关键词=子宫附件炎) OR 题名或关键词=子宫内膜炎) OR 题名或关键词=子宫内膜炎症) OR 题名或关键词=卵巢炎) OR 题名或关键词=子宫旁组织炎) OR 题名或关键词=盆腔蜂窝织炎) OR 题名或关键词=输卵管炎) OR 题名或关键词=盆腔炎) AND ((((((((((((((((((((((((((((((((((题名或关键词=针灸 OR 题名或关键词=针灸疗法) OR 题名或关键词=针刺疗法) OR 题名或关键词=针刺) OR 题名或关键词=针法) OR 题名或关键词=火针疗法) OR 题名或关键词=毫火针疗法) OR 题名或关键词=火针) OR 题名或关键词=毫火针) OR 题名或关键词=穴位注射) OR 题名或关键词=穴位注射疗法) OR 题名或关键词=微针疗法) OR 题名或关键词=耳针疗法) OR 题名或关键词=耳针) OR 题名或关键词=头针疗法) OR 题名或关键词=头针) OR 题名或关键词=浮针疗法) OR 题名或关键词=浮针) OR 题名或关键词=腹针疗法) OR 题名或关键词=腹针) OR 题名或关键词=头皮针) OR 题名或关键词=体针疗法) OR 题名或关键词=体针) OR 题名或关键词=毫针) OR 题名或关键词=灸法) OR 题名或关键词=艾灸) OR 题名或关键词=温针) OR 题名或关键词=温针灸) OR 题名或关键词=温针疗法) OR 题名或关键词=隔物灸) OR 题名或关键词=穴位疗法) OR 题名或关键词=穴位埋线) OR 题名或关键词=埋线) OR 题名或关键词=耳穴) OR 题名或关键词=针药并用)) AND (((((((((题名或关键词=随机对照 OR 题名或关键词=随机) OR 题名或关键词=对照) OR 题名或关键词=RCT) OR 题名或关键词=临床研究) OR 题名或关键词=疗效观察) OR 题名或关键词=临床观察) OR 题名或关键词=疗效) OR 题名或关键词=观察) OR 题名或关键词=临床))

**WanFang**

主题:(盆腔炎性疾病 OR 子宫附件炎 OR 子宫内膜炎 OR 子宫内膜炎症 OR 卵巢炎 OR 子宫旁组织炎 OR 盆腔蜂窝织炎 OR 输卵管炎 OR 盆腔炎) and 主题:(针灸 OR 针灸疗法 OR 针刺疗法 OR 针刺 OR 针法 OR 火针疗法 OR 毫火针疗法 OR 火针 OR 毫火针 OR 穴位注射 OR 穴位注射疗法 OR 微针疗法 OR 耳针疗法 OR 耳针 OR 灸法 OR 艾灸 OR 温针 OR 温针灸 OR 温针疗法 OR 隔物灸 OR 穴位疗法 OR 穴位埋线 OR 埋线 OR 耳穴 OR 针药并用) and 主题:(随机对照 OR 随机 OR 对照 OR RCT OR 临床研究 OR 疗效观察 OR 临床观察 OR 疗效 OR 观察 OR 临床)
