# Supplementary material for: Acupuncture therapies for relieving pain in pelvic inflammatory disease: A systematic review and meta-analysis
Source: PLoS One. 2024 Jan 31;19(1):e0292166. doi: 10.1371/journal.pone.0292166 (PMC10830011; doi:10.1371/journal.pone.0292166)
Supplement: S2 Appendix — (PDF) [file pone.0292166.s003.pdf]

Author(s):
Question: Acupuncture therapies compared to Routine treatments for relieving pain in PID
Setting:
Bibliography:

Certainty assessment							№ of patients		Effect		Certainty	Importance
**№ of studies**	**Study design**	**Risk of bias**	**Inconsistency**	**Indirectness**	**Imprecision**	**Other considerations**	**Acupuncture therapies**	**Routine treatments**	**Relative (95% CI)**	**Absolute (95% CI)**		
**VAS score for abdominal pain**												
12	randomised trials	serious[a]	not serious	not serious	serious[b]	publication bias strongly suspected[c]	582	583	-	MD **1.32 lower** (1.6 lower to 1.05 lower)	⊕◯◯◯ Very low	CRITICAL
**VAS score for lumbosacral pain**												
3	randomised trials	serious[a]	not serious	not serious	serious[d]	none	186	186	-	MD **1.14 lower** (2.12 lower to 0.17 lower)	⊕⊕◯◯ Low	CRITICAL
**VAS score for abdominal pain after one month**												
3	randomised trials	serious[a]	not serious	not serious	serious[d]	none	90	91	-	MD **1.44 lower** (2.15 lower to 0.72 lower)	⊕⊕◯◯ Low	CRITICAL
**IL-2**												
3	randomised trials	serious[a]	not serious	not serious	serious[d]	none	121	121	-	SMD **1.6 SD higher** (1.31 higher to 1.89 higher)	⊕⊕◯◯ Low	IMPORTANT
**IL-6**												
2	randomised trials	serious[a]	serious[e]	not serious	serious[d]	none	110	110	-	SMD **2.59 SD lower** (3.61 lower to 157 lower)	⊕◯◯◯ Very low	IMPORTANT
**TNF-α**												
3	randomised trials	serious[a]	not serious	not serious	serious[d]	none	182	182	-	SMD **2.21 SD lower** (2.47 lower to 1.95 lower)	⊕⊕◯◯ Low	IMPORTANT
**CRP**												
3	randomised trials	serious[a]	not serious	not serious	serious[d]	none	179	179	-	MD **3.85 lower** (5.15 lower to 2.63 lower)	⊕⊕◯◯ Low	IMPORTANT
**SAS**												
2	randomised trials	serious[a]	serious[e]	not serious	serious[d]	none	103	103	-	MD **10.82 lower** (16.6 lower to 5.04 lower)	⊕◯◯◯ Very low	IMPORTANT
**WHOQOL-BREF**												
2	randomised trials	serious[a]	serious[e]	not serious	serious[d]	none	114	114	-	MD **8.29 higher** (3.48 higher to 13.1 higher)	⊕◯◯◯ Very low	IMPORTANT
**Adverse events**												
3	randomised trials	serious[a]	not serious	not serious	serious[d]	none	11/175 (6.3%)	20/175 (11.4%)	**OR 0.56** (0.21 to 1.51)	**47 fewer per 1,000** (from 88 fewer to 49 more)	⊕⊕◯◯ Low	CRITICAL

CI: confidence interval; **MD:** mean difference; **OR:** odds ratio; **SMD:** standardised mean difference

Explanations

a. Methodological quality of included studies was not high.
b. The pooled (cumulative) sample size was lower than the optimal information size (OIS).
c. Publication bias detected by funnle plots.
d. The total enrollment was less than 400.
e. The pooled outcome revealed unexplained high heterogeneity.
